# Pathogenic bacteria features of central line-associated bloodstream infections in ICU patients: focus on the early predictive value of neutrophil-to-lymphocyte and platelet-to-lymphocyte ratios

**DOI:** 10.3389/fcimb.2025.1525758

**Published:** 2025-04-30

**Authors:** Yuzhen Li, Yanyan Li, Jiqin Wang, Keyu Sun

**Affiliations:** Department of Emergency, Minhang Hospital, Fudan University, Shanghai, China

**Keywords:** central line-associated bloodstream infection (CLABSI), pathogenic bacteria, drug resistance, neutrophil to lymphocyte ratio(NLR), platelet to lymphocyte ratio(PLR), procalcitonin(PCT)

## Abstract

**Objective:**

Explore and analyze CLABSI pathogenic bacteria characteristics in ICU patients and the value of PCT, NLR, PLR in early infection prediction.

**Methods:**

926 ICU patients with central venous catheters in Minhang Hospital from January 2021 to December 2023 were enrolled. They were grouped by co-infection status. PCT, NLR and PLR levels were measured, patient data analyzed, pathogenic bacteria characteristics summarized, and their predictive value evaluated via ROC curve.

**Results:**

From January 2021 to December 2023, among the 926 patients with CVC, 73 were diagnosed with CLABSI, with an infection rate of 7.88%. A total of 81 strains of pathogenic bacteria were isolated, including 60.50% (49/81) Gram - positive bacteria, 35.80% (29/81) Gram - negative bacteria, and 3.70% (3/81) fungi. The main Gram - positive bacteria exhibited high resistance to penicillin, erythromycin, clindamycin, and oxacillin, with a resistance rate exceeding 70%, yet were sensitive to vancomycin, linezolid, and tetracycline. The main Gram - negative bacteria had high resistance to piperacillin, piperacillin/tazobactam, Aztreonam, and gentamicin, with a resistance rate over 70%, and were more sensitive to cefoperazone/sulbactam, imipenem, and amikacin. Age, the site of catheterization, the duration of catheterization, and the employment of double - cavity catheters were all factors that exerted an influence on CLABSI among ICU patients (with p < 0.05). The levels of peripheral blood NLR, PLR, and PCT in the infected group were higher than those in the non - infected group (p < 0.05). The areas under the curve (AUCs) of peripheral blood NLR, PLR, and PCT were 0.814, 0.798, and 0.856, respectively, with the largest AUC for PCT. When the cut - off point was 2.75 ng/ml, the Youden index was the largest. The AUCs of the combination of peripheral blood NLR and PLR, NLR and PCT, PLR and PCT, and all three combined were 0.877, 0.903, 0.857, and 0.917.

**Conclusion:**

The early prediction of CLABSI in ICU patients by means of PCT, NLR, and PLR is of remarkable significance. It can provide a precious reference for clinical diagnostic and treatment strategies.

## 1 Introduction

As a commonly used diagnosis and treatment method in intensive care units (ICUs), central venous catheters (CVC) are widely used in hemodynamic monitoring, parenteral nutrition, hemofiltration, fluid resuscitation, etc. However, as a foreign body, it is easily colonized by microorganisms, which may lead to central line-associated bloodstream infection (CLABSI), thereby increasing mortality, morbidity and healthcare costs (van der Kooi et al., 2023; Teja et al., 2024; Xu et al., 2024).

Patients with CLABSI typically present without characteristic clinical features. Generally, fever is the most prominent initial sign. Redness, swelling, pain, and suppuration at the intubation site are highly specific manifestations. Other clinical presentations include hemodynamic instability, catheter malfunction, and sudden-onset sepsis, among others (Singer et al., 2016). If the infection fails to be effectively managed, it can give rise to severe complications, thereby impacting the patients’ quality of life, prolonging hospitalization, and even resulting in death (Badia-Cebada et al., 2022). At present, bacterial culture remains the gold - standard for diagnosing CLABSI. Nevertheless, its low positive culture rate and long processing time limit its guiding value in early - stage clinical treatment (Liu et al., 2021; Ruiz-Ruigómez and Aguado, 2021). The neutrophil to lymphocyte ratio (NLR) and platelet to lymphocyte ratio (PLR) are effective indicators in blood biochemical indicators that can evaluate the inflammatory state of the body. The detection is simple and easy to obtain. Currently, they have been applied in the diagnosis and prognosis of diseases including systemic lupus erythematosus and influenza (Luo et al., 2022; Zinellu et al., 2022). Serum procalcitonin (PCT) is widely applied in bacterial infectious diseases. It is not influenced by other diseases and has relatively high diagnostic specificity, and its value has already been recognized (Zhang et al., 2022; Bajić et al., 2023). This study analyzed the pathogenic bacteria and drug - resistance status of patients with CLABSI and explored the diagnostic value of peripheral blood NLR, PLR, and PCT for such infections, aiming to provide a reference for early clinical diagnosis and treatment.

## 2 Subjects and methods

### 2.1 Subjects

**Figure 1:**
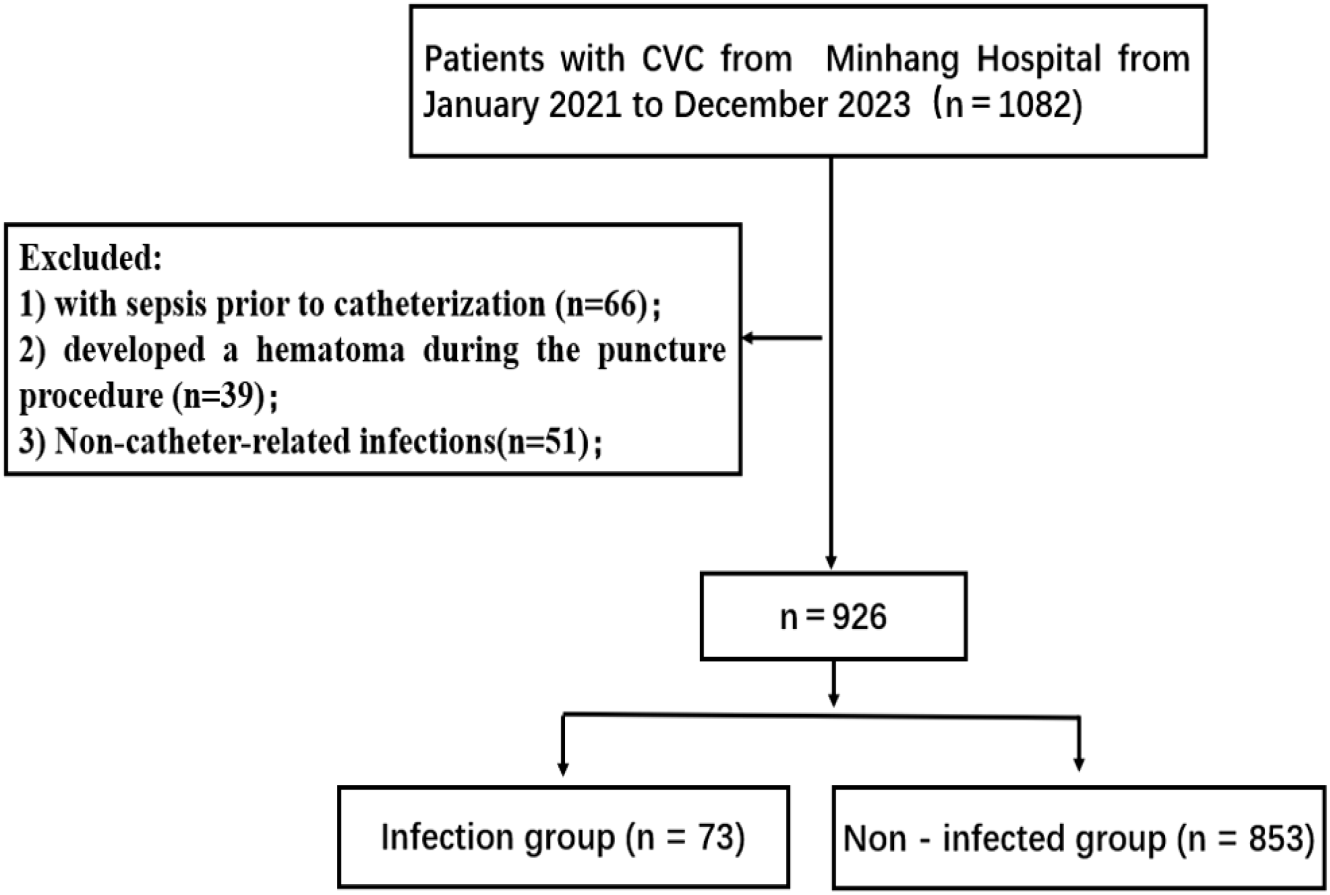
The flow of patient selection.

A total of 926 patients with CVC in the ICU of the Minhang Hospital were enrolled as the research subjects. Inclusion criteria were as follows (**Figure 1**): (1) Patients with CVC within the ICU of the hospital; (2) Those having indwelling CVC for over 48 hours; (3) In the situation of multiple catheter - related infections in one patient, only the first instance was chosen; (4) Patients with reports of microbiological examinations related to the catheter. Exclusion criteria were: (1) Patients who had been clearly diagnosed with sepsis prior to catheterization; (2) Patients who developed a hematoma during the puncture procedure; (3) Nosocomial infections due to non - catheter - associated factors. The diagnostic criteria for central venous catheter - related infections referred to the Clinical Practice Guidelines for the Diagnosis and Management of CLABSI in the United States (Hentrich et al., 2014). This study was reviewed and approved by the hospital’s medical ethics committee.

### 2.2 Methods

Cases were retrieved through the hospital’s medical record query system. Two staff members were assigned to fill in the form, while another two were responsible for the review process. The statistical content covered various aspects: patients’ basic information (including gender, age, body mass index, and past medical history), details of the catheter (such as type, indwelling site, duration of indwelling, and the time interval from catheter insertion to the onset of infection), results of pathogen detection and drug - sensitivity tests. For the infected group, the NLR, PLR, and PCT values were selected within 48 hours before blood culture and catheter culture were performed when infection was suspected. For patients in the non - infected group, the results of blood routine examinations and PCT tests were selected on the day of CVC removal. Blood routine examination items include neutrophils, platelets, and lymphocytes, among others. Based on these elements, the NLR and PLR are calculated.

### 2.3 Statistical analysis

The SPSS21.0 software was employed for data analysis. The counting data were expressed in the form of case numbers or percentages, and either the *χ*² test or the Fisher’s exact probability method was utilized. Measurements following a normal distribution were presented as (x̄ ± s), while those with a non - normal distribution were presented as P_50_ (P_25_, P_75_). An independent - sample T - test was conducted for inter - group comparison. The receiver operating characteristic (ROC) curve of subject characteristics was drawn to analyze the value of serological indices in diagnosing CLABSI. The test level α = 0.05 for both - sided tests, and a p - value less than 0.05 was considered statistically significant.

## 3 Results

### 3.1 Infection rate and related conditions

A total of 926 patients with a central venous catheter in the ICU were investigated from January 2021 to December 2023. Among them, 73 patients developed CLABSI, resulting in an infection rate of 7.88%. Among the infected patients, 45 were male and 28 were female. Fifty patients were 60 years old or older, while 23 were younger than 60. There were 30 patients with a catheter indwelling time of 14 days or more, and 43 patients with a catheter indwelling time of less than 14 days. Multiple logistic regression analysis indicated that age, catheterization site, catheterization time, and the application of a double - luminal catheter were independent influencing factors for CLABSI (P < 0.05), as presented in **Tables 1**, **2**.

**Table 1:** Univariate analysis of the CLABSI.

Project	Infected Group (n = 73)	Non-infected group(n = 853)	*χ*² - value	*P* - value
Gender (Male/Female)	45/28	465/388	1.382	0.240
Age (in years)	61.18 ± 8.39	60.59 ± 8.82	0.579	0.577
BMI (kg/m²)	21.73 ± 3.84	22.40 ± 3.65	0.896	0.462
Catheter Indwelling Time (days)			6.280	0.012
<14d	43	620		
≥14d	30	233		
Indwelling Catheter Position			7.476	0.006
Non - femoral Vein	28	469		
Femoral Vein	45	384		
Use of Double - lumen Catheters			7.868	0.005
Yes	39	314		
No	34	539		
History of Diabetes Mellitus			3.212	0.073
No	54	703		
Yes	19	150		

BMI, body mass index.

**Table 2:** Multivariate logistic regression analysis of the infection in CLABSI.

Associated Factors and Variable Assignment	*β*	*S.E*	*Wald χ²*	*P - value*	*OR*	*95%CI*
Age	1.965	0.827	4.968	0.029	6.287	2.891, 37.834
Tube Placement Location	3.217	0.587	11.223	0.012	14.926	3.897, 52.437
Indwelling Time of Catheter	1.447	0.430	12.320	0.007	4.503	1.116, 19.228
Use of Double - lumen Catheters	3.762	0.536	11.874	0.009	15.327	4.396, 59.671

### 3.2 Distribution of pathogenic bacteria

**Figure 2:**
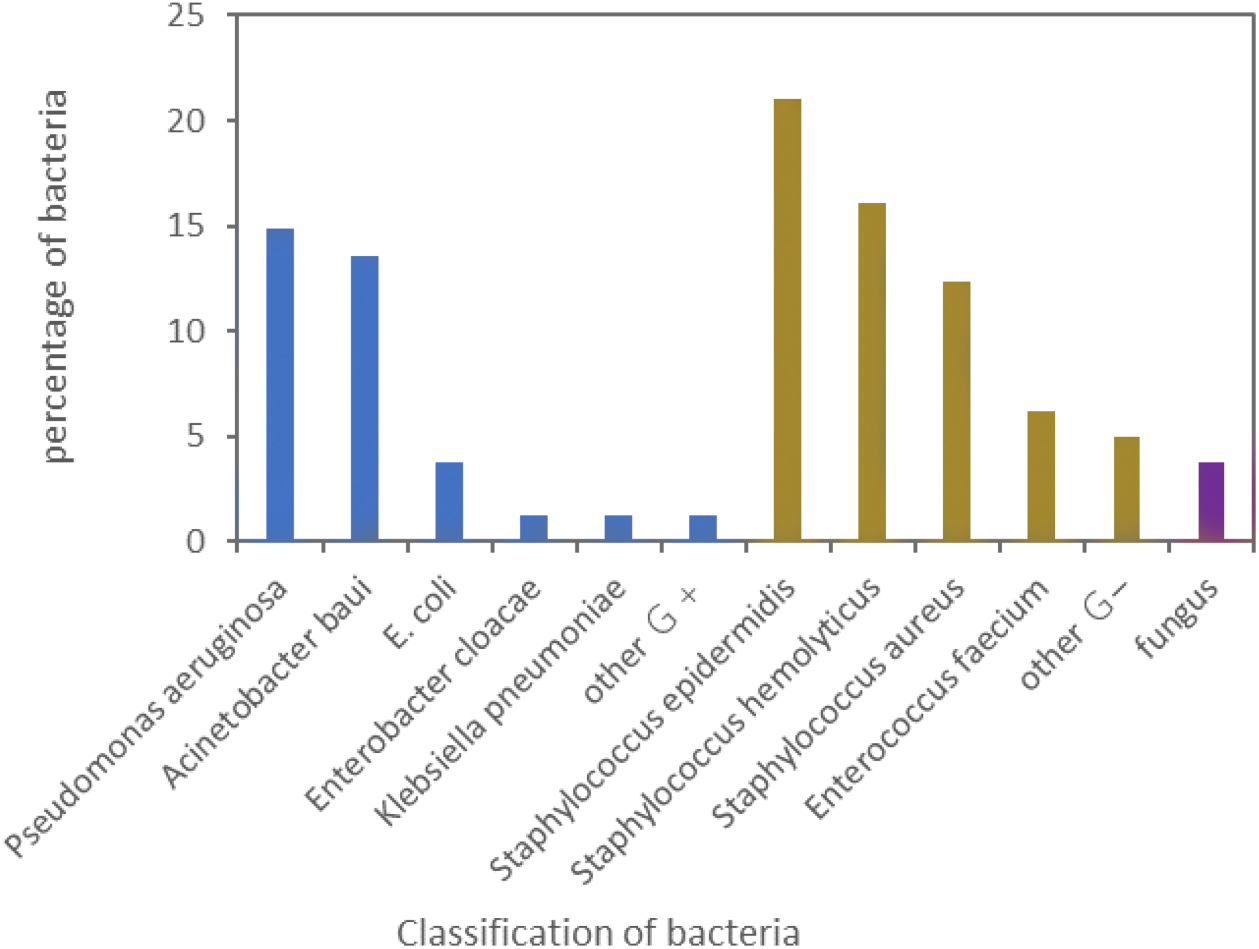
Constituent ratio of the pathogens isolated from the patients with CLABSI.

In this study, 73 patients with CLABSI had 81 pathogenic bacteria strains detected. Gram - positive bacteria accounted for 60.50% (49/81), gram - negative ones for 35.80% (29/81), and fungi for 3.70% (3/81). The specific proportion of each is shown in **Figure 2**.

### 3.3 Drug resistance of major Gram - positive bacteria

Among the pathogens related to CLABSI in the intensive care unit, gram - positive bacteria exhibited high resistance to penicillin, erythromycin, clindamycin, and oxacillin, with a resistance rate exceeding 70%. In contrast, vancomycin, linezolid, and tetracycline demonstrated low drug resistance, having a resistance rate of less than 20%, as presented in **Table 3**.

**Table 3:** Drug Resistance of Major Gram - positive Bacteria in CLABSI in the Intensive Care Unit [Strains (%)].

Antibacterial drug	Staphylococcus aureus (n = 10)		Staphylococcus hemolyticus (n = 13)		Staphylococcus epidermidis (n = 17)	
	N	Drug Resistance Rate (%)	N	Drug Resistance Rate (%)	N	Drug Resistance Rate (%)
Levofloxacin	3	30.00	5	38.46	6	35.29
Ciprofloxacin	5	50.00	8	61.54	7	41.18
Linezolid	1	10.00	2	15.38	0	0.00
Oxacillin	9	90.00	12	92.31	16	94.12
Vancomycin	0	0.00	0	0.00	0	0.00
Clindamycin	8	80.00	11	84.62	14	82.35
Tetracycline	4	40.00	4	30.77	9	52.94
Erythromycin	9	90.00	12	92.31	16	94.12
Penicillin	8	80.00	10	76.92	15	88.24

### 3.4 Drug resistance of major Gram - negative bacteria

Among the pathogens of CLABSI in intensive care units, the principal Gram - negative bacteria exhibited high resistance to cefepime, piperacillin, aztreonam, and gentamicin, with the drug resistance rate exceeding 70%. In contrast, cefoperazone/sulbactam, imipenem, and amikacin demonstrated low drug resistance, having resistance rates lower than 20%, as presented in **Table 4**.

**Table 4:** Drug resistance of major Gram-negative bacteria in CLABSI in intensive care unit [strains (%)].

Antibacterial Drug	Acinetobacter baumannii (n = 11)		Pseudomonas aeruginosa (n = 12)	
	N	Drug Resistance Rate (%)	N	Drug Resistance Rate (%)
Levofloxacin	6	54.55	6	50.00
Ciprofloxacin	5	45.45	5	41.67
Amikacin	2	18.18	2	16.67
Gentamicin	9	81.82	9	75.00
Meropenem	4	36.36	5	41.67
Imipenem	0	0.00	0	0.00
Aztreonam	8	72.73	9	75.00
Piperacillin	9	81.82	11	91.67
Cefoperazone - Sulbactam	1	9.09	1	8.33
Cefotaxime	6	54.55	8	66.67
Cefepime	10	90.91	11	91.67
Ceftazidime	5	45.45	8	66.67

### 3.5 The levels of peripheral blood PCT, NLR, and PLR of patients in both the infected and non - infected groups were compared

The peripheral blood PCT levels of patients in the infected and non - infected groups differed statistically (P < 0.001). When the NLR and PLR levels of the two groups were compared, it was found that the NLR and PLR in the infected group were significantly higher than those in the non - infected group (p < 0.001) (**Table 5**).

**Table 5:** Peripheral Blood PCT, NLR and PLR Levels in Patients of the Infected and Uninfected Groups.

Index	Non-infected Group (n = 853)	Infected Group (n = 73)	*t* value	*P* value
PCT, median (Q1, Q3)	0.43 (0.29, 0.67)	5.87 (4.83, 7.70)	-30.024	<0.001
PLR, median (Q1, Q3)	121.71 (98.04, 141.79)	168.35 (143.24, 217.39)	-10.177	<0.001
NLR, median (Q1, Q3)	2.28 (1.87, 2.96)	6.80 (2.90, 9.60)	-8.827	<0.001

### 3.6 Value of peripheral blood PCT, NLR, and PLR levels in the diagnosis of CLABSI individually and in combination

**Figure 3:**
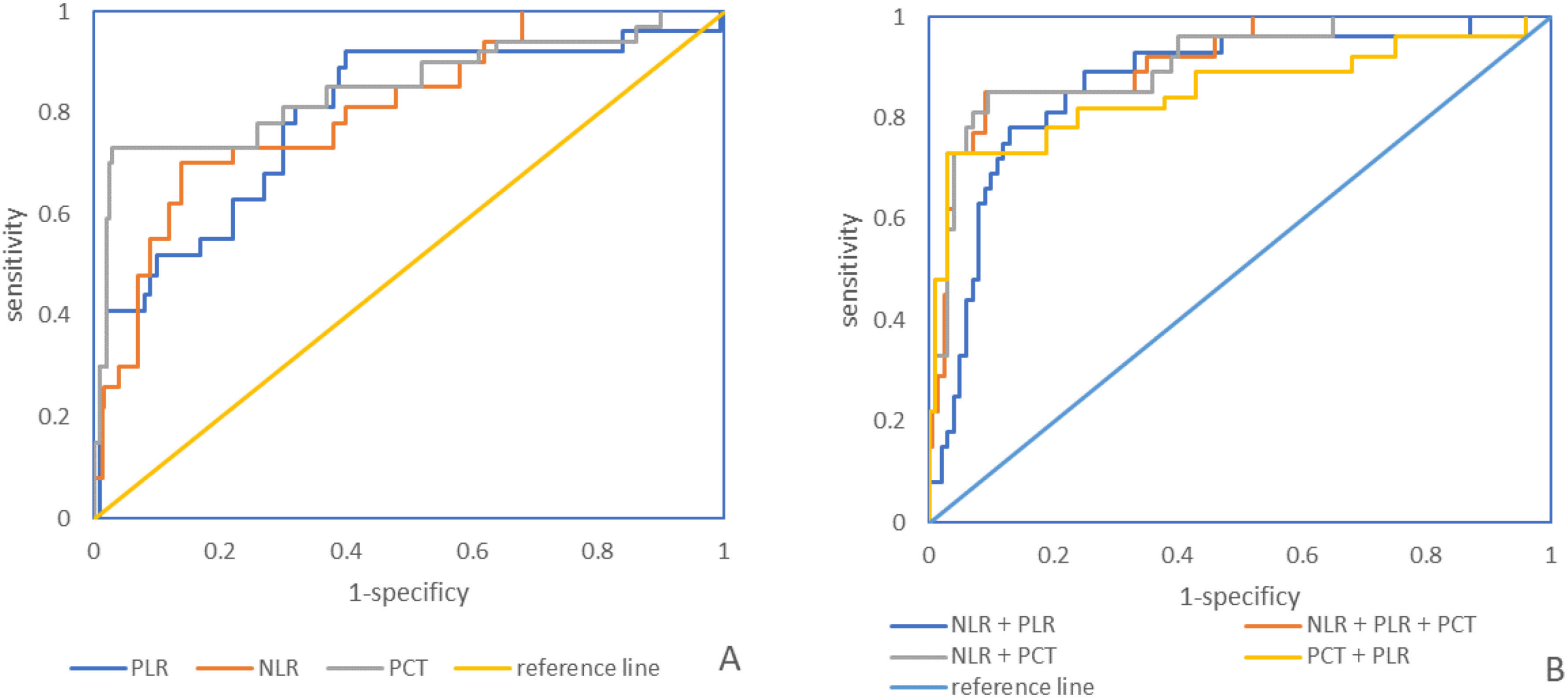
**(A)**. ROC curve of peripheral blood PCT, NLR, and PLR levels individually for the diagnosis of CLABSI; **(B)**. ROC curve of peripheral blood PCT, NLR, and PLR levels in combination for the diagnosis of CLABSI.

The AUC of the levels of PCT, NLR, and PLR in peripheral blood for diagnosing CLABSI were 0.856, 0.814, and 0.798 respectively. The AUC for PCT diagnosis was the largest. When the cut - off point was 2.75 ng/ml, the Youden index reached its maximum. In the combined diagnosis, the AUCs of peripheral blood PLR combined with NLR, PLR combined with PCT, NLR combined with PCT, and the combination of all three in diagnosing CLABSI were 0.877, 0.857, 0.903, and 0.917 respectively. Among these combinations, the combination of all three had the highest diagnostic efficiency, as presented in **Table 6** and **Figure 3**.

**Table 6:** Diagnostic value of peripheral blood PCT, NLR and PLR in CLABSI.

Index	AUC	Cut - off value	Sensitivity (%)	Specificity (%)	95% *CI*	*P* value
PCT	0.856	2.75	74.14	96.53	0.762-0.949	<0.001
PLR	0.798	130.24	92.66	50.92	0.700-0.895	<0.001
NLR	0.814	2.87	81.52	62.27	0.724-0.901	<0.001
PCT+NLR	0.903	一	85.27	91.36	0.849-0.975	<0.001
PCT+PLR	0.857	一	81.55	76.27	0.757-0.954	<0.001
PLR+NLR	0.877	一	88.93	75.07	0.804-0.948	<0.001
PCT+NLR+PLR	0.917	一	85.26	92.90	0.827-0.978	<0.001

## 4 Discussion

In this study, it has been determined that the risk factors for CLABSI within ICUs include an age of ≥60 years, femoral vein catheterization, a catheter placement time of ≥14 days, and the use of double - lumen catheters. This finding coincides with certain research on CLABSI (Chua et al., 2014; Cheng et al., 2019; de Grooth et al., 2020; Pitiriga et al., 2020). It suggests that the patient’s age, the site of catheterization, the duration, and the type of catheter all increase the incidence of CLABSI in ICUs to varying degrees. Consequently, for clinical catheterization, the internal jugular vein or subclavian vein should be selected whenever feasible. Additionally, the catheter should be removed as soon as possible, and the single - cavity catheter should be preferred.

In this study, through analyzing the distribution of pathogens related to CLABSI, it was found that among 926 patients in the ICU, 73 suffered from CLABSI, with an infection rate of 7.88%. In the United States, approximately 16,000 cases of CLABSI occur in ICUs, and around 400 - 5,000 patients die annually due to catheter infection (Chua et al., 2014). In this research, a total of 81 strains of pathogenic bacteria were identified in 73 patients with CLABSI. Among them, gram - positive bacteria accounted for 60.50%, gram - negative bacteria for 35.80%, and fungi for 3.70%. The most common gram - positive bacteria was Staphylococcus epidermidis (20.99%), and the most prevalent gram - negative bacteria was Pseudomonas aeruginosa (14.82%). Pinto et al. (2022) investigated patients in three large hospitals and revealed that the positive rate of Staphylococcus epidermidis was 36.21%, and among gram - negative bacteria, the positive rate of Pseudomonas aeruginosa was the highest. This is similar to the results of this study and relevant literature (Barrigah-Benissan et al., 2023; Rha et al., 2023; Tabah et al., 2023), indicating that gram - positive bacteria are the principal pathogens in patients with CLABSI in the ICU, which has guiding significance for clinical drug use.

In the present study, through the monitoring of the drug resistance profiles of the principal pathogens in patients diagnosed with Central Venous Catheter-Related Bloodstream Infections (CLABSI), several notable findings were obtained. It was observed that Gram-positive bacteria manifested a high level of resistance to penicillin, erythromycin, clindamycin, and oxacillin, with the resistance rates surpassing 70%. In contrast, their resistance to vancomycin, linezolid, and tetracycline was relatively low, as evidenced by the resistance rates remaining below 20%. Regarding Gram-negative bacteria, these pathogens demonstrated a pronounced resistance to cefepime, piperacillin, aztreonam, and gentamicin, where the resistance rates exceeded 70%. However, they exhibited a low resistance to Cefoperazone-Sulbactam, imipenem, and amikacin, with the resistance rates being less than 20%. Previous studies (Ohnuma et al., 2023) have suggested that vancomycin could be considered as the preferred therapeutic option for treating Gram-positive bacterial infections in the context of CLABSI, especially in regions characterized by a low prevalence of CLABSI and a relatively low susceptibility to vancomycin. Concurrently, within the scope of this study, it was found that the resistance rate of amoxicillin in the drug-susceptibility testing was the highest among the antibiotics tested, followed by that of ampicillin. For Gram-negative bacteria, imipenem was identified as the antibiotic to which they exhibited the highest sensitivity, followed by tobramycin. Nevertheless, it should be noted that these Gram-negative bacteria still displayed a relatively high resistance rate to aztreonam and ceftriaxone. This comprehensive understanding of the drug resistance patterns of the main pathogens in CLABSI patients can provide valuable insights for guiding the rational selection of antibiotics in clinical practice, aiming to improve the treatment outcomes and mitigate the impact of antibiotic resistance.

In this study, NLR, PLR, and PCT were selected as predictors for CLABSI. NLR serves as an indicator reflecting the levels of neutrophils and lymphocytes within the body. During sepsis and other systemic infections, the activation of extracellular regulatory kinase and other substances can be observed, and the enhanced expression of anti - apoptotic proteins leads to the delay of neutrophil apoptosis. A higher count of neutrophils implies a more severe inflammatory response within the body and a more intense systemic inflammatory reaction. Meanwhile, the large consumption of lymphocytes can directly reflect the degree of inflammation in the body and provide auxiliary evidence for the early diagnosis of bacterial bloodstream infections. (Buonacera et al., 2022; Li et al., 2022; Wang et al., 2023). PLR is an index that reflects the levels of platelets and lymphocytes. Besides their hemostatic and thrombosis - promoting functions, platelets also possess a certain pathogen - killing effect. In the process of systemic inflammatory response, endotoxin can trigger platelet activation and the secretion of pro - inflammatory factors such as chemokines and nitric oxide. These molecules attach to and bind with neutrophils, activate them, and result in the production of reactive oxygen species, cytokines, and other substances to eliminate pathogenic microorganisms. Hence, the level of endotoxin can also indicate the degree of inflammation in the body (Kim et al., 2019; Kriplani et al., 2022; Kearney et al., 2023). The results of this study demonstrated that NLR and PLR were significantly elevated in the infected group compared to the uninfected group, indicating that the levels of NLR and PLR increase with CLABSI. PCT, a protein consisting of 116 amino acid residues, is a precursor of calcitonin. Studies (Lee et al., 2022; Bajić et al., 2023; Oussalah et al., 2023) have revealed that PCT can be released in large quantities during infectious diseases, and its appearance time precedes that of other cytokines, enabling it to better reflect the body’s inflammatory response syndrome. The results of this study indicated that patients with CLABSI had a substantial increase in PCT, which was significantly higher than that of the uninfected group. In this study, the diagnostic AUC of each index was > 0.7, validating that each index has significant value in the early diagnosis of CLABSI. Among the indicators in this study, the PCT level has the highest diagnostic value. The possible reason is that routine blood indexes such as neutrophils, lymphocytes, and platelets can be influenced by numerous factors, whereas the PCT level only changes significantly in the context of infectious diseases, endowing it with higher diagnostic specificity and efficacy for infectious diseases (Centor and Gilbert, 2022; Schuetz, 2022). In this study, the combined - diagnosis AUC of multiple indicators was greater than that of any single indicator. Therefore, the combined detection of multiple indicators can be used to enhance the diagnostic efficiency of CLABSI in clinical practice.

The limitations of this paper are summarized as follows: (1) Sample size limitation: The study included only 73 cases in the infection group (infection rate: 7.88%), which is relatively small and may affect statistical power and the stability of results. (2) Limitations of retrospective design: The study is based on retrospective data, which may be subject to selection bias (e.g., non-randomized grouping), information bias (e.g., incomplete data recording), or inadequate control of confounding factors (e.g., lack of detailed records on antibiotic use history and immunosuppressive status). (3) External validity of single-center study: The research was conducted exclusively at Minhang Hospital. Results may be influenced by specific medical environments, operational norms, or regional pathogen distributions, making it difficult to generalize to other medical institutions or regions.

CLABSI in ICU requires close attention. Key risk factors include age, catheter site, insertion duration, and double-cavity catheter use. Gram-positive bacteria are the primary pathogens, and targeted antibiotic therapy based on microbial characteristics is critical for effective treatment. Elevated peripheral blood NLR, PLR, and PCT levels can aid CLABSI diagnosis (individually or combined), guiding early intervention and improving outcomes.

## Data Availability

The original contributions presented in the study are included in the article/supplementary material. Further inquiries can be directed to the corresponding authors.

## References

[B1] Badia-Cebada L. Peñafiel J. Saliba P. Andrés M. Càmara J. Domenech D.. (2022). Trends in the epidemiology of catheter-related bloodstream infections; towards a paradigm shift, Spain, 2007 to 2019. Euro Surveill. 27, 2100610. doi: 10.2807/1560-7917.ES.2022.27.19.2100610 35551704 PMC9101967

[B2] Bajić D. Matijašević J. Andrijević L. Zarić B. Lalić-Popović M. Andrijević I.. (2023). Prognostic role of monocyte distribution width, CRP, procalcitonin and lactate as sepsis biomarkers in critically ill COVID-19 patients. J. Clin. Med. 12, 1197. doi: 10.3390/jcm12031197 36769843 PMC9917557

[B3] Barrigah-Benissan K. Ory J. Simon C. Loubet P. Martin A. Beregi J. P.. (2023). Clinical factors associated with peripherally inserted central catheters (PICC) related bloodstream infections: a single centre retrospective cohort. Antimicrob Resist. Infect. Control. 12, 5. doi: 10.1186/s13756-023-01209-z 36717942 PMC9885663

[B4] Buonacera A. Stancanelli B. Colaci M. Malatino L. (2022). Neutrophil to lymphocyte ratio: an emerging marker of the relationships between the immune system and diseases. Int. J. Mol. Sci. 23, 3636. doi: 10.3390/ijms23073636 35408994 PMC8998851

[B5] Centor R. M. Gilbert D. N. (2022). Annals on call - procalcitonin in the diagnosis of bacterial infection. Ann. Intern Med. 175, OC1. doi: 10.7326/A21-0006 35226519

[B6] Cheng S. Xu S. Guo J. He Q. Li A. Huang L.. (2019). Risk factors of central venous catheter-related bloodstream infection for continuous renal replacement therapy in kidney intensive care unit patients. Blood Purif. 48, 175–182. doi: 10.1159/000495024 30485840

[B7] Chua H. R. Schneider A. G. Sherry N. L. Lotfy N. Chan M. J. Galtieri J.. (2014). Initial and extended use of femoral versus nonfemoral double-lumen vascular catheters and catheter-related infection during continuous renal replacement therapy. Am. J. Kidney Dis. 64, 909–917. doi: 10.1053/j.ajkd.2014.04.022 24882583

[B8] de Grooth H. J. Timsit J. F. Mermel L. Mimoz O. Buetti N. du Cheyron D.. (2020). Validity of surrogate endpoints assessing central venous catheter-related infection: evidence from individual- and study-level analyses. Clin. Microbiol Infect. 26, 563–571. doi: 10.1016/j.cmi.2019.09.022 31586658

[B9] Hentrich M. Schalk E. Schmidt-Hieber M. Chaberny I. Mousset S. Buchheidt D.. (2014). Central venous catheter-related infections in hematology and oncology: 2012 updated guidelines on diagnosis, management and prevention by the Infectious Diseases Working Party of the German Society of Hematology and Medical Oncology. Ann. Oncol. 25, 936–947. doi: 10.1093/annonc/mdt545 24399078

[B10] Kearney N. McCourt C. Hambly R. Hughes R. O’Kane D. Kirby B. (2023). Association of biologic treatment in hidradenitis suppurativa with reduced neutrophil-lymphocyte ratio and platelet-lymphocyte ratio. JAMA Dermatol. 159, 222–224. doi: 10.1001/jamadermatol.2022.5710 36576747 PMC9857331

[B11] Kim Y. J. Kang J. Ryoo S. M. Ahn S. Huh J. W. Kim W. Y.. (2019). Platelet-lymphocyte ratio after granulocyte colony stimulating factor administration: an early prognostic marker in septic shock patients with chemotherapy-induced febrile neutropenia. Shock. 52, 160–165. doi: 10.1097/SHK.0000000000001256 30148761

[B12] Kriplani A. Pandit S. Chawla A. de la Rosette J. J. M. C. H. Laguna P. Jayadeva Reddy S.. (2022). Neutrophil-lymphocyte ratio (NLR), platelet-lymphocyte ratio (PLR) and lymphocyte-monocyte ratio (LMR) in predicting systemic inflammatory response syndrome (SIRS) and sepsis after percutaneous nephrolithotomy (PNL). Urolithiasis. 50, 341–348. doi: 10.1007/s00240-022-01319-0 35246692 PMC9110452

[B13] Lee E. H. Lee K. H. Song Y. G. Han S. H.. (2022). Discrepancy of C-reactive protein, procalcitonin and interleukin-6 at hospitalization: infection in patients with normal C-reactive protein, procalcitonin and high interleukin-6 values. J. Clin. Med. 11, 7324. doi: 10.3390/jcm11247324 36555941 PMC9783053

[B14] Li D. Li J. Zhao C. Liao X. Liu L. Xie L.. (2022). Diagnostic value of procalcitonin, hypersensitive C-reactive protein and neutrophil-to-lymphocyte ratio for bloodstream infections in pediatric tumor patients. Clin. Chem. Lab. Med. 61, 366–376. doi: 10.1515/cclm-2022-0801 36367370

[B15] Liu B. M. Carlisle C. P. Fisher M. A. Shakir S. M.. (2021). The brief case: capnocytophaga sputigena bacteremia in a 94-year-old male with type 2 diabetes mellitus, pancytopenia, and bronchopneumonia. J. Clin. Microbiol. 59, e0247220. doi: 10.1128/JCM.02472-20 34142857 PMC8218739

[B16] Luo S. Yang W. S. Shen Y. Q. Chen P. Zhang S. Q. Jia Z.. (2022). The clinical value of neutrophil-to-lymphocyte ratio, platelet-to-lymphocyte ratio, and D-dimer-to-fibrinogen ratio for predicting pneumonia and poor outcomes in patients with acute intracerebral hemorrhage. Front. Immunol. 13, 1037255. doi: 10.3389/fimmu.2022.1037255 36300107 PMC9589455

[B17] Ohnuma T. Chihara S. Costin B. Treggiari M. M. Bartz R. R. Raghunathan K.. (2023). Association of appropriate empirical antimicrobial therapy with in-hospital mortality in patients with bloodstream infections in the US. JAMA Netw Open 6, e2249353. doi: 10.1001/jamanetworkopen.2022.49353 36598788 PMC9857618

[B18] Oussalah A. Callet J. Manteaux A. E. Thilly N. Jay N. Guéant J. L.. (2023). Usefulness of procalcitonin at admission as a risk-stratifying biomarker for 50-day in-hospital mortality among patients with community-acquired bloodstream infection: an observational cohort study. Biomark Res. 11, 4. doi: 10.1186/s40364-023-00450-3 36647149 PMC9843889

[B19] Pinto M. Borges V. Nascimento M. Martins F. Pessanha M. A. Faria I.. (2022). Insights on catheter-related bloodstream infections: a prospective observational study on the catheter colonization and multidrug resistance. J. Hosp Infect. 123, 43–51. doi: 10.1016/j.jhin.2022.01.025 35189301

[B20] Pitiriga V. Kanellopoulos P. Bakalis I. Kampos E. Sagris I. Saroglou G.. (2020). Central venous catheter-related bloodstream infection and colonization: the impact of insertion site and distribution of multidrug-resistant pathogens. Antimicrob Resist. Infect. Control. 9, 189. doi: 10.1186/s13756-020-00851-1 33261661 PMC7708904

[B21] Rha B. See I. Dunham L. Kutty P. K. Moccia L. Apata I. W.. (2023). Vital signs: health disparities in hemodialysis-associated staphylococcus aureus bloodstream infections - United States, 2017-2020. MMWR Morb Mortal Wkly Rep. 72, 153–159. doi: 10.15585/mmwr.mm7206e1 36757874 PMC9925139

[B22] Ruiz-Ruigómez M. Aguado J. M. (2021). Duration of antibiotic therapy in central venous catheter-related bloodstream infection due to Gram-negative bacilli. Curr. Opin. Infect. Dis. 34, 681–685. doi: 10.1097/QCO.0000000000000763 34261908

[B23] Schuetz P. (2022). How to best use procalcitonin to diagnose infections and manage antibiotic treatment. Clin. Chem. Lab. Med. 61 (5), 822-828. doi: 10.1515/cclm-2022-1072 36317790

[B24] Singer M. Deutschman C. S. Seymour C. W. Shankar-Hari M. Annane D. Bauer M.. (2016). The third international consensus definitions for sepsis and septic shock (Sepsis-3). JAMA. 315, 801–810. doi: 10.1001/jama.2016.0287 26903338 PMC4968574

[B25] Tabah A. Buetti N. Staiquly Q. Ruckly S. Akova M. Aslan A. T.. (2023). Epidemiology and outcomes of hospital-acquired bloodstream infections in intensive care unit patients: the EUROBACT-2 international cohort study. Intensive Care Med. 49, 178–190. doi: 10.1007/s00134-022-06944-2 36764959 PMC9916499

[B26] Teja B. Bosch N. A. Diep C. Pereira T. V. Mauricio P. Sklar M. C.. (2024). Complication rates of central venous catheters: A systematic review and meta-analysis. JAMA Intern Med. 184, 474–482. doi: 10.1001/jamainternmed.2023.8232 38436976 PMC12285596

[B27] van der Kooi T. I. I. Smid E. A. Koek M. B. G. Geerlings S. E. Bode L. G. M. Hopmans T. E. M.. (2023). The effect of an intervention bundle to prevent central venous catheter-related bloodstream infection in a national programme in the Netherlands. J. Hosp Infect. 131, 194–202. doi: 10.1016/j.jhin.2022.11.006 36414165

[B28] Wang R. H. Wen W. X. Jiang Z. P. Chen P. Zhang S. Q. Jia Z.. (2023). The clinical value of neutrophil-to-lymphocyte ratio (NLR), systemic immune-inflammation index (SII), platelet-to-lymphocyte ratio (PLR) and systemic inflammation response index (SIRI) for predicting the occurrence and severity of pneumonia in patients with intracerebral hemorrhage. Front. Immunol. 14, 1115031. doi: 10.3389/fimmu.2023.1115031 36860868 PMC9969881

[B29] Xu H. Hyun A. Mihala G. Rickard C. M. Cooke M. L. Lin F.. (2024). The effectiveness of dressings and securement devices to prevent central venous catheter-associated complications: A systematic review and meta-analysis. Int. J. Nurs. Stud. 149, 104620. doi: 10.1016/j.ijnurstu.2023.104620 37879273

[B30] Zhang R. M. Tan K. Fu S. Deng J. K. (2022). Limited value of procalcitonin, C-reactive protein, white blood cell, and neutrophil in detecting bacterial coinfection and guiding antibiotic use among children with enterovirus infection. World J. Pediatr. 18, 230–233. doi: 10.1007/s12519-021-00504-2 35061203 PMC8898256

[B31] Zinellu A. Zinellu E. Mangoni A. A. Pau M. C. Carru C. Pirina P.. (2022). Clinical significance of the neutrophil-to-lymphocyte ratio and platelet-to-lymphocyte ratio in acute exacerbations of COPD: present and future. Eur. Respir. Rev. 31, 220095. doi: 10.1183/16000617.0095-2022 36323421 PMC9724880

